# *rox*: A Statistical Model for Regression with Missing Values

**DOI:** 10.3390/metabo13010127

**Published:** 2023-01-13

**Authors:** Mustafa Buyukozkan, Elisa Benedetti, Jan Krumsiek

**Affiliations:** Institute for Computational Biomedicine, Department of Physiology and Biophysics Weill Cornell Medicine, New York, NY 10021, USA

**Keywords:** missing values, regression analysis, limit-of-detection

## Abstract

High-dimensional omics datasets frequently contain missing data points, which typically occur due to concentrations below the limit of detection (LOD) of the profiling platform. The presence of such missing values significantly limits downstream statistical analysis and result interpretation. Two common techniques to deal with this issue include the removal of samples with missing values and imputation approaches that substitute the missing measurements with reasonable estimates. Both approaches, however, suffer from various shortcomings and pitfalls. In this paper, we present “rox”, a novel statistical model for the analysis of omics data with missing values without the need for imputation. The model directly incorporates missing values as “low” concentrations into the calculation. We show the superiority of rox over common approaches on simulated data and on six metabolomics datasets. Fully leveraging the information contained in LOD-based missing values, rox provides a powerful tool for the statistical analysis of omics data.

## 1. Introduction

**Figure 1:**
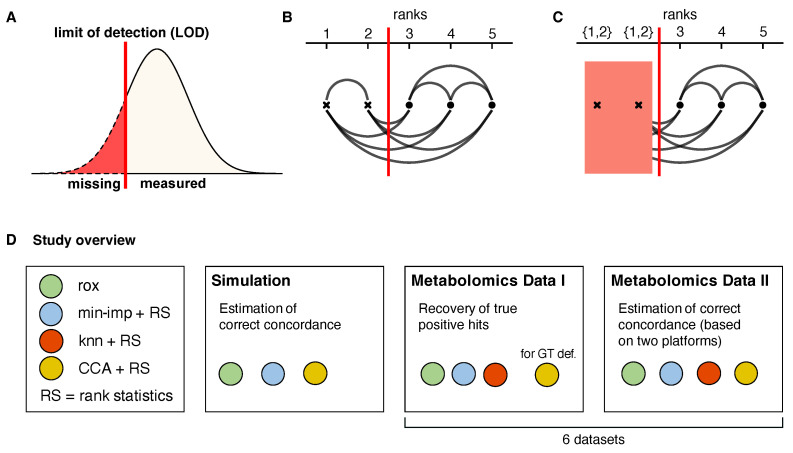
**Limit of detection (LOD)-based missingness, statistical concept, and study overview.** (**A**): Schematic of a strict LOD effect on the distribution of a measurement. Values below the LOD (red line) will be reported as missing. (**B**): Relative order of data points based on their true value. The red line indicates the theoretical LOD. (**C**): Observed ordering of data points after LOD censoring. While observations below the LOD (red line) cannot be compared once they are censored, we still retain the information that all points below the LOD are lower than all points above the LOD. (**D**): Overview of rox benchmarking. We assess the performance of our approach using an extensive simulation framework, followed by two test scenarios of ground-truth recovery on a series of metabolomics datasets. “for GT def.” = used for definition of ground truth.

High-dimensional molecular datasets, such as metabolomics, proteomics, glycomics, and microbiomics, typically contain a substantial amount of “missing values”, that is, measurement points for which the experimental platform did not return any quantified value [1,2,3]. Any analysis workflow applied to data with missing values needs to deal with this issue, since most common statistical approaches do not allow for the absence of data points. Missing values in omics data usually occur due to abundances below the instrument sensitivity, the so-called limit of detection (LOD) [3] (Figure 1A). In addition to the obvious loss of information, the presence of missing values interferes with distributional assumptions for statistical analysis. For example, metabolomics measurements are generally log-normally distributed [4], and therefore LOD-based missing values will obfuscate the lower tail of the distribution. In microbiome data, which are compositional in nature [5], left truncation will lead to an artificial overrepresentation of the most abundant species. Further complicating the issue, we have previously shown that LOD effects are not always strict and can occur in blurry fashion, where lower concentration values increase the chance of a value being reported as missing rather than depending on a strict threshold [3].

A statistical method for the analysis of molecular data should take into consideration the abovementioned issues and properties of missing values. First, it should make use of the fact that a missing value indicates a “low” abundance value, even if the precise numeric value is unknown. This allows fully leveraging the information available in the dataset. Ideally, the method should also work in the presence of a non-strict LOD mechanism. Second, in order to be applicable to a wide variety of molecular data, the method should be free of distributional assumptions and robust to outliers.

Existing statistical methods dealing with LOD-based missing values do not or only partially fulfill these requirements. The most popular approaches fall into one of three categories. (1) Missing values are simply deleted from the dataset, which is commonly referred to as complete case analysis (CCA) [6]. Since all samples with any missing values are removed, CCA often leads to a severe reduction in the statistical power, especially when multivariate statistical methods are used. Moreover, if there is an enrichment of missing values in one of the analyzed groups, e.g., in sick individuals compared to healthy ones, CCA will substantially distort the statistical analysis and produce erroneous results [3]. (2) Imputation approaches reconstruct a full data matrix by replacing missing values with reasonable substitutes. “Minimum imputation” is a widely used approach that replaces missing values with the lowest observed value in the data, half of that value, or with a known LOD value [3]. Notably, this approach uses the information that missing values are low but leads to a substantial distortion of the distribution of the analyte [7]. Other common approaches, such as k-nearest-neighbor (knn) imputation, use the correlation structure of the data to infer the original value [8]. These approaches do not use the LOD information and require a strong correlation structure among variables to work properly. (3) The third approach is to use statistical methods that directly incorporate the knowledge of the LOD effect, where missing values are treated as a “low” category. The approach published by [9] addresses the problem of LOD-based left censoring in measurement data using methods from survival analysis, which we prove later in this paper is equivalent to using rank statistics on minimum imputed data. Other approaches make specific assumptions about the underlying data distribution (e.g., log-normal [10] or gamma [11]), and treat the missing values as left-truncated data points from that respective distribution. While incorporating the LOD information, these methods also require strong assumptions on the overall data distribution, which might not be appropriate for certain data types.

Here we present rox, “rank order with missing values(X)”, a flexible, nonparametric approach for regression analysis of a dependent variable with missing values and continuous, ordinal, or binary explanatory variables. The core idea is to utilize the knowledge of missing values representing low concentrations due an LOD effect, without requiring any actual imputation steps. The approach is based on rank statistics related to Somer’s D and Kendall’s tau [12,13,14], and can be computed even with partially quantitative measurements (Figure 1B,C). Leveraging the properties of rank statistics, this framework is applicable to data from any distribution and is robust to outliers. Moreover, while the method relies on the assumption of an LOD effect in its core, it flexibly generalizes to data with other missingness mechanisms.

In this paper, we showcase the features of rox on simulated data and benchmark its performance on six real molecular datasets. We use metabolomics data, which is known to be heavily affected by LOD-based missingness and therefore constitutes an optimal test case for this approach. Notably, both for the simulated data and the real data, we define a ground truth for unbiased evaluation. Our analysis demonstrates the superiority of our approach over three of the most commonly used approaches in the field, namely complete case analysis (CCA), minimum imputation and knn-based imputation, coupled with rank-based statistical testing (Figure 1D). Our rox implementation is available as open source R package at https://github.com/krumsieklab/rox.

## 2. Methods

### 2.1. rox Core Model

rox is inspired by the ranking-based, nonparametric correlation measure *concordance index* or c-index [15], which is equivalent to an ROC-AUC with a binary outcome [16]. Let $S=\{\left(x_{1},y_{1}\right),\ldots ,\left(x_{n},y_{n}\right)\}$ be a set of *n* observations of two random variables *X* and *Y*. A pair of observations $\langle i,j\rangle =\left\{\left(x_{i},y_{i}\right),\left(x_{j},y_{j}\right)\right\}$ is said to be *concordant* if the pairwise ranking of $\left(x_{i},x_{j}\right)$ and $\left(y_{i},y_{j}\right)$ is the same, i.e., if $\mathrm{sgn}\left(x_{i}-x_{j}\right)=\mathrm{sgn}\left(y_{i}-y_{j}\right)$; otherwise, it is said to be *discordant*. The c-index is then defined as the number of concordant pairs over the number of all pairs $c_{YX}=\frac{\mathrm{concordant}}{\mathrm{concordant}+\mathrm{discordant}}=\frac{\mathrm{concordant}}{\mathrm{all}\;\mathrm{pairs}}$[15]. Note that in $c_{YX}$, the ties in *Y* are dropped from the calculation, whereas in *X*, they are counted as 0.5, i.e., neither concordant nor discordant.

The rox statistic is an extension of this c-index concept to left-censored data. This type of data occurs, for example, when all values below a certain threshold (the *Limit Of Detection*, or LOD) are returned as missing. Based on this LOD assumption, we know that any missing value is lower than any measured value in the data. Importantly, a missing and a measured value can thus still be ranked and are hence *comparable*, while two missing values have no known order and are *noncomparable*. The rox method assesses the fraction of concordant pairs only in relation to the *comparable pairs*, $\frac{\mathrm{concordant}}{\mathrm{comparable}\;\mathrm{pairs}}$, a concept that is also used in survival analysis [15]. In the following, we formulate a concordance-based test on comparable pairs.

Let *Y* now be a left-censored random variable with LOD-based missing values, and let *X* be an outcome of interest to be associated with *Y*. As outlined above, any pair of observations 〈i,j〉 where at least one of $y_{i}$ or $y_{j}$ is non-missing constitutes a comparable pair, since we know that the LOD requires, by design, any non-missing value to be larger than all missing values (see also, Figure 1B,C). Let $\pi =\{\langle i,j\rangle |y_{i}\neq \mathrm{NA}\mathrm{or}y_{j}\neq \mathrm{NA}\}$ be the set of these comparable pairs, where NA represents a missing value, and let Γ(π) be the number of concordant pairs in π given *X* and *Y*. The nonparametric rox coefficient of concordance between variables *X* and *Y*, with *Y* subject to LOD-based missingness, can be formulated as follows:

(1) $$rox_{core}=\frac{\Gamma (\pi )}{\left|\pi \right|}\mathrm{with}\Gamma \left(\pi \right)=\sum_{\langle i,j\rangle \in \pi }1_{\mathrm{if}\;\mathrm{sgn}\left(x_{i}-x_{j}\right)=\mathrm{sgn}\left(y_{i}-y_{j}\right)}$$

where |π| is the set size of π, i.e., the total number of comparable pairs.

In general, the sgn operator is not defined for missing values. However, under strict-LOD assumptions, any missing value in *Y* will be lower than all non-missing values. This means that $\mathrm{sgn}\left(y_{i}-y_{j}\right)$ is always defined in this framework, even when either $y_{i}$ or $y_{j}$ is missing. Note that, similar to the c-index, the rox statistic represents the probability of concordance between *X* and *Y*, and it can take values in the interval [0,1], where 0.5 indicates random ordering, 1 represents perfect concordance, and 0 represents perfect discordance.

### 2.2. Debiased Weighted Rox Model

In Equation (1), pairs where both $y_{i}$ and $y_{j}$ are missing constitute noncomparable pairs, which are excluded from the concordance estimation. However, ignoring these pairs leads to an overall overestimation of positive concordance (>0.5) and an underestimation of negative concordance (<0.5); see remark at the bottom of Supplementary Text S2. Note that the 0.5 cut point is due to the scale of concordance between 0 and 1, where values above 0.5 indicate positive correlation and values below 0.5 represent negative correlation.

To address this problem, we propose a strategy to debias the rox coefficient by downweighting the contribution of missing observations to the overall concordance. To this end, we split all comparable pairs from Equation (1) into two distinct sets: $\pi_{b}$ (bridge pairs), which includes pairs of observations where either $y_{i}$ or $y_{j}$ is missing, and $\pi_{1}$, which includes pairs where both $y_{i}$ and $y_{j}$ are non-missing (see Supplementary Figure S6). This way, all comparable pairs are partitioned as $\pi =\pi_{b}\cup \pi_{1}$.

With this formulation, we can now introduce a weight parameter *p* to control the contribution of the pairs with missing values, $\pi_{b}$, to the overall rox statistics as:

(2) $$rox=\frac{\Gamma \left(\pi_{1}\right)+p\Gamma \left(\pi_{b}\right)}{|\pi_{1}\left|+p\right|\pi_{b}|},$$

where 0≤p≤1 (see Supplementary Text S1 for a detailed derivation). Setting p=1 leads to the original formulation from Equation (1), which is based on a strict LOD assumption, whereas p=0 reduces the test statistics to a nonparametric complete case analysis, ignoring the contribution of all pairs including any missing values.

In general, if $n_{0}$ is the number of missing values, $n_{1}$ is the number of non-missing values, and $n=n_{0}+n_{1}$ is the total number of observations, a higher fraction of missing values $n_{0}/n$ will introduce more bias into the concordance estimation and hence require a lower value of the weight *p* to debias the estimate. In Supplementary Text S3, we demonstrate that the concordance can be effectively debiased by using the weight factor $p=n_{1}/n$. With this new expression for *p*, Equation (2) becomes:

(3) $$rox=\frac{\Gamma \left(\pi_{1}\right)+\frac{n_{1}}{n}\Gamma \left(\pi_{b}\right)}{|\pi_{1}|+\frac{n_{1}}{n}\left|\pi_{b}\right|}$$

### 2.3. Self-Adjusting Rox for Partial LOD and Non-LOD

The weighted formulation in Equation (3) assumes that the missingness in *Y* occurs due to a strict LOD threshold, i.e., that *all* values below the LOD threshold will be missing and *all* values above the threshold will be present. However, in many real data scenarios, missingness patterns occur on a continuum [3], from a strict LOD mechanism, to a more probabilistic setting, where lower values have a higher likelihood of being missing, all the way to missing-at-random (MAR). For the rox statistic, non LOD-based missingness constitutes a source of bias that will affect the estimation of the true concordance. For cases where the missingness pattern is only marginally due to LOD or even LOD-independent, ignoring the missing values and switching to a complete case analysis is more appropriate.

We thus formulated a self-adjusting version of rox. First, we estimated whether the missingness pattern in the data was consistent with an LOD assumption. Let $d_{1}=\Gamma \left(\pi_{1}\right)/\left|\pi_{1}\right|$ and $d_{b}=\Gamma \left(\pi_{b}\right)/\left|\pi_{b}\right|$ be the concordances of pairs with no missing values and pairs with one missing value, respectively. Under strict LOD, which corresponds to a left truncation of the data distribution, if the true concordance is larger than 0.5, then it holds that $d_{1}<d_{b}$, while for concordance values less than 0.5 it holds that $d_{b}<d_{1}$ (see Supplementary Text S2 for proof). For simplicity, we only describe the positive concordance case here; the negative concordance case can be derived analogously.

For any random variable *Y*, we can assess whether the LOD assumption is violated by checking whether $d_{1}<d_{b}$. If the inequality holds, rox concordance is estimated using Equation (3); if it does not, *p* in Equation (2) is set to zero, removing all missing observations from the analysis and effectively computing the concordance based only on the observations with no missing values, reducing the approach to a complete-case-analysis (CCA).

Thus, the final formulation of rox for positive concordance is:

(4) $$rox=\frac{\Gamma \left(\pi_{1}\right)+\Gamma \left(\pi_{b}\right)p_{{}_{d_{1}<d_{b}}}}{|\pi_{1}\left|+\right|\pi_{b}|p_{{}_{d_{1}<d_{b}}}}$$

where $p_{{}_{d_{1}<d_{b}}}=p=n_{1}/n$, as in Equation (3), if $d_{1}<d_{b}$; otherwise, it is 0.

### 2.4. Rox-Based Semiparametric Multivariable Model

The rox model handles one-to-one relations between two variables. In this section, we extend the approach to a semiparametric multivariable modeling framework, which allows modeling the relations between one variable with missing values and multiple variables.

The proposed extension is obtained via the multivariable modeling of the concordance probabilities with an exponential link function [17]. Let *Y* be a metabolite measurement with missing values and $X=\{X_{1},X_{2},\ldots ,X_{k}\}$ be *k* different variables of interest. First, we define the likelihood of concordance for a single pair of observations 〈i,j〉 as $L_{\langle i,j\rangle }=P\left(y_{i}<y_{j}|\langle i,j\rangle \right)=e^{\eta_{i}}/\left(e^{\eta_{i}}+e^{\eta_{j}}\right)$, where $\eta_{i}=\beta_{1}x_{i1}+\beta_{2}x_{i2}+\ldots +\beta_{k}x_{ik}$ is the standard linear predictor function for sample *i*, and $\beta =\{\beta_{1},\beta_{2},\ldots ,\beta_{k}\}$ is the vector of the corresponding regression coefficients. The log-likelihood ℓ(π) of the associated joint probability of all realized pairwise rankings in π can then be formulated as the product of all individual likelihoods:

(5) $$\ell \left(\pi \right)=\log\left(L\left(\pi \right)\right)=\log\left(\prod_{\forall \langle i,j\rangle \in \pi }L_{\langle i,j\rangle }\right)=\sum_{\forall \langle i,j\rangle \in \pi }\left(\eta_{i}-\log\left(e^{\eta_{i}}+e^{\eta_{j}}\right)\right).$$

Similar to the univariate case, Equation (5) ignores pairs of observations where both *Y* values are missing. However, ignoring these observations leads to a biased estimate of the concordance probability. In this case, we can also debias the model by downweighting the contribution of the missing values and accounting for non-LOD scenarios. We again partition the observation pairs in π into those between two observations with no missing value $\pi_{1}$ and those where one of the two observations is missing $\pi_{b}$ (i.e. $\pi =\pi_{1}\cup \pi_{b}$), we introduce a weight *p* to control the contribution of pairs with missing values, and we check for the violation of the LOD assumption based on the $d_{1}<d_{b}$ inequality:

(6) $$\ell_{rox}\left(\pi =\pi_{1}\cup \pi_{b}\right)=\ell \left(\pi_{1}\right)+p_{{}_{d_{1}<d_{b}}}\ell \left(\pi_{b}\right),$$

where $p=n_{1}/n$, as derived in Supplementary Text S3. All β coefficients are fitted using a maximum likelihood estimation (MLE) approach, based on a FORTRAN implementation for concordance regression adapted from [17]. The overall concordance of the model is then calculated by computing the rox statistic from Equation (4) between *Y* and the fitted score $\hat{\eta }=X\hat{\beta }$.

### 2.5. Hypothesis Testing

In the following, we derive a hypothesis test for the univariate version of rox, assessing whether $H_{0}:rox\left(Y,X\right)=0.5$ can be rejected. Under the null hypothesis of the independence of *Y* and *X*, the distribution of the quantity 2×rox−1 has an expected value of zero. A significance test for rox can be obtained via: $z=\left(rox-0.5\right)/\sqrt{Var(rox)}$, and the corresponding *p*-value can be calculated via a z-test. The variance of the concordance can be estimated in two different ways: (1) using an equivalent time-dependent Cox model (cvar) [16] or (2) through an unbiased infinitesimal jackknife variance estimator (ivar) [18]. As pointed out by Therneau et al. [18], cvar is an unbiased estimator for d=0.5, while it overestimates the variance if d≠0.5. On the other hand, ivar is unbiased for d≠0.5, but it underestimates the variance if *d* is close to 0.5. Taking these findings into consideration, we calculate the *p*-value of the estimated rox statistics based on the average of these two variance estimates, namely, $z=\left(rox-0.5\right)/\sqrt{(cvar+ivar)/2}$. This approach was inspired by [19], where overestimated and underestimated variances were averaged to obtain a better estimate.

In a multivariable setting, hypothesis testing for the overall model is performed as described in the previous paragraph, with $H_{0}:rox\left(Y,X\beta \right)=0.5$, where $\beta =\{\beta_{1},\beta_{2},\ldots ,\beta_{k}\}$ are the regression coefficients, and Xβ is the linear predictor of the model. Furthermore, to test the significance of individual variables in the model, we can use the coefficients in the proposed semiparametric model. In this case, the null hypothesis is defined based on the coefficients: $H_{0}:\beta_{i}=0$ for variable *i*. To assess the significance, we used the implementation of [17] to estimate the coefficients and standard errors to calculate a Wald’s test [20].

### 2.6. Simulation Framework

Two continuous variables *Y* and *X* with predefined concordance values were simulated. However, concordance cannot be directly parameterized and needs to be determined empirically. Here, we generated the desired concordance by tuning an association parameter as follows: The variable *X* and a noise term ϵ were first sampled from a standard normal distribution. *Y* was then defined as Y=X+λ·ϵ, where λ determines the association between *Y* and *X*. Larger values of λ lead to lower concordance between the two variables. We ranged λ from 0 to 0.7 in steps of 0.01 until the desired concordance d(Y,X) was reached.

For the large sample size simulation, we generated a total of n=10,000 samples. For the small sample size simulation, we first generated a large dataset of n=1,000,000 samples, from which 100 random samples were drawn 1000 times.

In the multivariable case, we simulated a variable *Y*, an outcome of interest *X*, and a covariate *Z*. Correlations between X,Y, and *Z* were simulated as follows: *X* was sampled from a normal distribution. A correlated covariate *Z* was simulated as $Z=X+\epsilon_{z}$, with $\epsilon_{z}$ being a normal distribution. Y was sampled as $Y=X+Z+3\epsilon_{y}$, with $\epsilon_{y}$ again being a normally distributed error term.

### 2.7. Metabolomics Datasets

To illustrate the performance of rox on real data, we analyzed a total of seven previously published metabolomics datasets (Table 1). For the QMDiab plasma validation cohort (HD4), only samples and metabolites overlapping with the HD2 platform were considered. Except for the HAPO dataset, for which only preprocessed data were available, all datasets were preprocessed using the R package maplet [21] as follows: Prior to the statistical analysis, the raw peak intensities were normalized using the probabilistic quotient approach [22], using only metabolites with less than 20% missing values to generate the reference sample. Normalized metabolite values were subsequently ${log}_{2}$ transformed. The following imputation step was applied for all datasets. For each cohort, two additional data matrices were generated: one where missing values were imputed using the minimum value per metabolite and one where missing values were imputed using knn-based imputation with 10 neighbors and variable preselection based on pairwise correlation (threshold of 0.2), according to [3].

## 3. Results

### 3.1. Simulation Results: Strict LOD

The rox method uses rank-based statistics to model measurements with limit of detection (LOD)-based missing value patterns, utilizing the information that absent data points represent low concentrations. It models the relationship between a measurement with missing values as the dependent variable and one or more continuous, ordinal, or binary explaining variables with no missing values. The approach furthermore implements a self-adjusting feature, which detects cases of non-LOD missingness, in which it switches to complete case analysis (CCA). A detailed mathematical derivation of the approach and its properties was provided in the Section 2.

**Figure 2:**
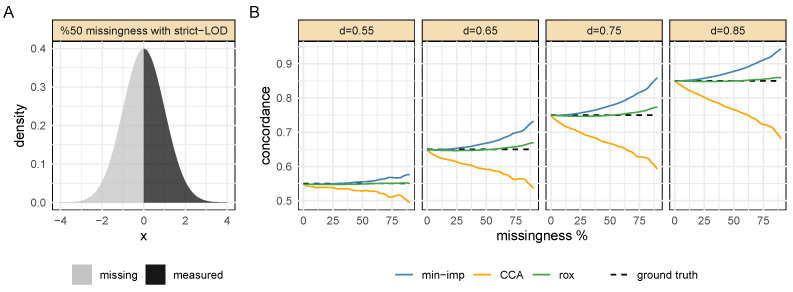
**Simulation with the strict LOD mechanism.** (**A**): Example distribution of a simulated variable with 50% missingness due to a strict LOD effect. (**B**): rox outperformed the CCA and minimum imputation in recovering the true concordance across various ground truth values *d* and missingness fractions. The minimum imputation led to an overestimation of the concordance between the variable and outcome, while the CCA resulted in an underestimation.

To show how rox performed at correctly recovering the true concordance, we developed an extensive simulation framework with a known ground truth. The performance of rox was compared to that of complete case analysis (CCA) and regular concordance calculation after minimum imputation (min-imp). Note that the k-nearest-neighbor (knn) imputation was omitted for this part, since it is only feasible in a multivariate setup, where simulation is dependent on various design choices and could easily be tweaked for a method to outperform the others. knn imputation is evaluated based on real datasets later. In the first simulation, a single variable *Y* and a continuous outcome *X* were simulated with the concordance *d* ranging between 0.55 and 0.85. These predefined concordance values represented the ground truth used to evaluate the performance. The missing values were introduced into *Y* using a strict LOD mechanism, i.e., by setting all values below a given threshold as missing (Figure 2A). We simulated a variety of scenarios by ranging the proportion of missing values in *Y* from 0% to 90%. For each combination of the true concordance and missing value proportion, we computed the concordance between *Y* and *X* by: (i) computing the rox statistic, (ii) imputing missing values in *Y* with minimum value imputation and performing regular concordance analysis, and (iii) only considering complete cases without missing values and performing regular concordance analysis. A large sample size of n= 10,000 was chosen to ensure stable results. All simulations were repeated for smaller sample sizes, which yielded equivalent results (see Supplementary Figure S1).

The results of this first simulation demonstrated that rox was consistently better at retrieving the true concordance than its two competitors (Figure 2B). The minimum imputation generally led to an overestimation of the concordance, while CCA led to an underestimation. With increasing proportions of the missing values, these deviations increased substantially, while the rox estimates remained stable and accurate. The same effect was observed across all values of the true concordance values *d*.

### 3.2. Simulation Results: Probabilistic LOD

**Figure 3:**
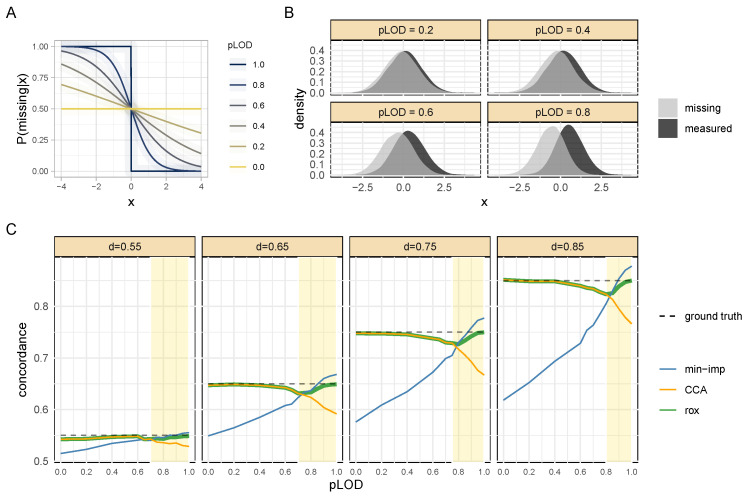
**Simulation with the probabilistic LOD mechanism**. (**A**): The probability function describing the likelihood of a value being missing as a function of its numerical value. A pLOD value of 1 results in a strict LOD effect, while pLod=0 results in values missing at random (MAR), and all values in between produce probabilistic LOD effects. (**B**): Illustration of the different missingness patterns induced by varying pLOD values. (**C**): The data were simulated with different pLOD and concordance values, while the missingness percentage was kept at 50% in all scenarios. The yellow shaded area marks the region where our adaptive method automatically identified an active LOD effect and switched from CCA to rox analysis. Overall, rox outperformed minimum imputation and complete case analysis in all simulation settings.

In the second simulation scenario, we evaluated the performance of rox in the case of a more realistic “probabilistic LOD” [3], where instead of a hard LOD threshold, the probability of a value being missing continuously increases with decreasing true abundance. A probabilistic LOD was simulated using a sigmoid probability density function that modeled the likelihood of a value being missing given its true value (Figure 3A). The shape of the sigmoid function was parametrized with a variable pLOD, which controlled the type of missingness pattern in the data. pLOD=0 led to missing at random (MAR) [29], while pLOD=1 generated a strict LOD effect. Therefore, higher values of pLOD led to more prominent censoring effects (Figure 3B). In this scenario, we again simulated two random variables *Y* and *X* with true concordance values ranging from d=0.55 to d=0.85 and pLOD values ranging from 0 to 1. For this setup, the proportion of missing values in *Y* was fixed at 50%.

The rox method again outperformed the competitor methods in all scenarios (Figure 3C) due to the adaptive nature of the model. For simulations with pLOD values below 0.7, rox determined that no sufficiently strong LOD effect was present and thus switched to complete case analysis (CCA). Minimum imputation, on the other hand, consistently underestimated the concordance in the range of *d* between 0 and 0.7, due to its strict implicit LOD assumption. For pLOD>0.7, rox leveraged the left-censoring effect and consistently produced more accurate results than its competitors. In this high pLOD range, minimum imputation overestimated the concordance, and the performance of the complete case analysis progressively deteriorated. This behavior was further exacerbated at increasing values of the true concordance *d*.

### 3.3. Simulation Results: Multivariable Setting

In a third simulation, we investigated how the rox model performed in a multivariable setting for both strict-LOD and probabilistic-LOD scenarios. The multivariate setting is of particular interest when the inclusion of multiple variables in the same model is required, for example for covariate correction purposes. In this scenario, we simulated a continuous outcome, a covariate, and a variable of interest under various LOD and missingness settings and again compared rox’s performance with that of minimum imputation and knn imputation followed by regular concordance analysis (see Supplementary Figure S2). The results and conclusions were equivalent to the results of univariate simulations suggesting that the rox recovered the ground truth better than the competing approaches.

### 3.4. Evaluation on Metabolomics Data: Recovering High-Confidence Hits

After evaluating the performance of rox in a simulation setting, we tested the approach in a real data scenario using published metabolomics datasets from a series of case-control studies (Table 1). In this case, we sought to determine how many true associations between metabolites and the respective study outcomes (e.g., disease status) rox could identify compared to its competitor methods.

Defining a ground truth in real datasets, however, is inherently difficult, since a list of true associations is usually not available. For our evaluation framework, we thus constructed a set of metabolite-outcome associations with high confidence of being actual true positives (HC-hits). This set was defined by combining the significant associations obtained from two statistical approaches that are well-suited to detect associations in data with antithetical missing values mechanisms. (1) CCA with the Wilcoxon rank-sum test, which compares two sample groups by filtering out all samples with missing values. This test works well if values are missing at random and thus do not originate from an LOD effect; however, it is generally underpowered since it entirely excludes missing values from the analysis. (2) Fisher’s exact test, which assesses the proportion of missing values in one sample group versus the other, ignoring the actual numeric measurement values. In an LOD setting, this test works well for cases of extreme sample separation, for example, when all samples in one of the comparison groups are low and fall below the LOD threshold. Notably, both approaches suffer from a substantial number of false negatives, since neither is ideally fit for the analysis of molecular data with missing values; however, both methods have very low false positive rates, meaning that the hits they identify are very likely to be correct.

**Figure 4:**
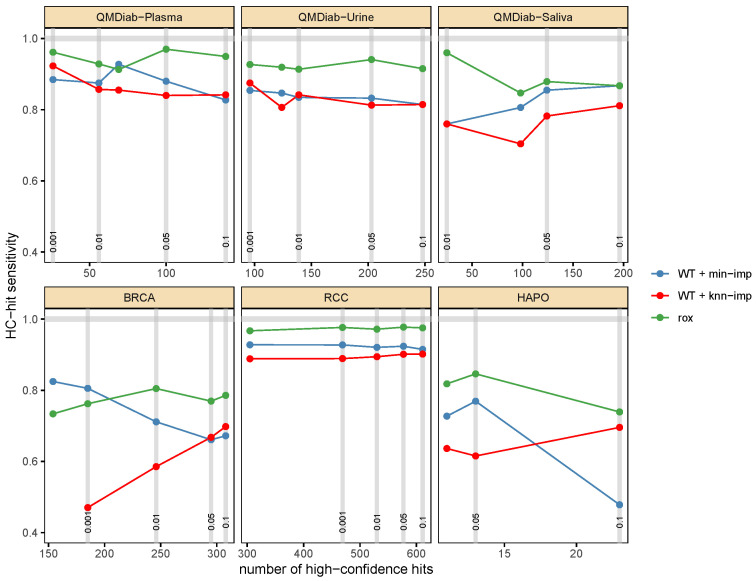
**Recovery of high-confidence (HC) hits in six metabolomics datasets**. The x-axis shows the number of HC-hits identified with the corresponding Bonferroni-adjusted *p*-value cutoff. WT: Wilcoxon rank-sum test. The y-axis represents the percentage of HC-hits that were identified, which is equivalent to a measure of sensitivity, at the respective cutoff. The rox outperformed or tied with the two imputation approaches across all datasets and cutoffs.

In the following, we used the fraction of HC-hits that each method was able to retrieve as an evaluation metric. The analysis was performed on six datasets: plasma, urine, saliva metabolomics from the QMDiab study [23], where the outcome was type-2-diabetes (T2D), a hyperglycemia study in pregnant women (HAPO) [26], with fasting plasma glucose (low FPG vs. high FPG) as the outcome, and two tissue metabolomics datasets, one from breast tissue (BRCA) [24] and one from kidney tissue (RCC) [25], where the outcome was the origin of the sample (tumor or adjacent-normal tissue) of the sample. Across all datasets, the rox outperformed or tied with the other methods in recovering the HC-hits at various significance levels (Figure 4).

### 3.5. Evaluation on Metabolomics Data: Multiplatform Validation

A second line of validation on real data was performed on a dataset from the QMdiab study, where the same metabolites were measured in the same samples using two different metabolomics platforms. Specifically, we analyzed metabolites that were fully quantified (FQ) on one platform but were partially missing (PM) on the other platform. We used the concordance between the study outcome and the FQ metabolites without missing values as our ground truth and evaluated the performance of each method based on the consistency between this ground truth and the concordance estimate between the PM metabolite with missing values and the same outcome. In an ideal scenario, these two concordance values would be the same, indicating that the method recovered the correct value even in the presence of missing values. To analyze a sufficient number of FQ–PM metabolite pairs, we allowed up to 5% missing values in the FQ candidate and deleted those missing values in the subsequent analysis. If both platforms showed less than 5% missingness for a metabolite, we picked the one with the lower number of missing values as the FQ metabolite and the respective other measurement as the PM metabolite.

Notably, the missing values in these platforms were mostly due to a prominent LOD effect, which we confirmed by comparing the missing and quantified values within the same metabolites across the two platforms (see Supplementary Figure S3). Thus, we expected this dataset to provide a favorable setting for minimum imputation, which assumes a strict LOD. knn imputation, on the other hand, cannot impute values outside of the observed data distribution and is therefore unlikely to perform well in a strong LOD scenario [30].

**Figure 5:**
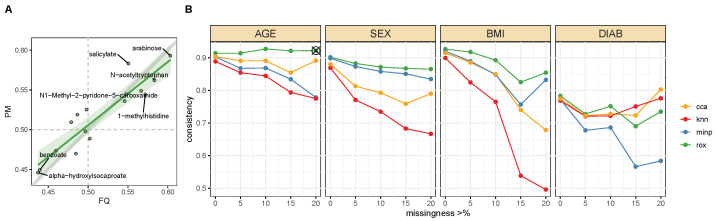
**Validation on the two-platform comparison of fully quantified (FQ) and partially missing (PM) metabolites**. Plasma metabolites from the same samples were measured on two different platforms. Consistency of the concordance estimates between the FQ-based ground truth and the PM-based estimates was computed for all considered outcomes (age, BMI, diabetes (DIAB), and sex). (**A**): Consistency across platforms for age associations calculated for PM metabolites with 20% or more missingness. Points distributed along the diagonal indicate consistent estimations between the two platforms. The gray line indicates the x = y axis, while the green line indicates a linear fit over the points of comparison. (**B**): Systematic results for all four outcomes and varying levels of missingness in the PM metabolites. The x-axis indicates the minimum fraction of missing values per metabolite. The y-axis shows the Pearson correlation between the estimates across the two platforms. Results shown in panel A correspond to the point marked with the black cross. Across all missingness percentages, rox was more consistent compared to knn imputation and comparable to or better than minimum imputation.

Association analyses were performed between both the FQ and PM metabolites and the respective QMdiab study outcomes (age, sex, BMI, and diabetes), using the rox test as well as regular association analysis with the Wilcoxon rank-sum test after min-imp, knn-imp, and CCA. The PM-based concordance values were then compared with the ground truth concordance obtained from the corresponding FQ metabolite. An example of the results for age and PM metabolites with 20% or more missingness is shown in Figure 5A. This analysis was systematically repeated for varying fractions of missingness in the PM metabolite (see Figure 5B and Supplementary Figure S4 for more detailed results). For all outcomes, rox was substantially more consistent than minimum imputation, regardless of missingness percentage of the PM metabolite. Notably, the performance of minimum imputation worsened with increasing missingness, while rox’s performance remained stable. The rox outperformed knn-imputation specifically in the association with age, sex, and BMI, while the two methods were mostly comparable for diabetes. Taken together, rox performed equivalent to or better than knn-imputation across all scenarios. Similar comparisons were performed using multivariable rox, with analogous results (see Supplementary Figure S5).

## 4. Discussion

This paper introduced rox, a novel statistical framework for datasets with missing values occurring due to a limit-of-detection (LOD) effect. In contrast to the more common approach of imputing missing values, which comes with various data analysis-related issues, rox directly utilized the information that missing values had “low” concentrations. The nonparametric model was based on pairwise ranks and was thus robust to outliers. The method allowed for multivariable modeling and can be used for any quantitative or semiquantitative measurements. Importantly, while rox was inherently designed for data with an LOD effect, it also worked with less strict blurry LOD-based data or even when the values were missing at random, in which case it automatically switched to complete case analysis. Using a simulation framework as well as metabolomics datasets from various sample types with different outcomes, we systematically demonstrated the superiority of our method over other approaches that are commonly used in the field. Specifically, rox showed higher accuracy in reconstructing the underlying true concordance values and displayed higher statistical power retrieving associations with study outcomes. Notably, while most other studies on real data artificially introduce missing values to evaluate the performance of their statistical approach (e.g., [8,31]), we relied on two data-driven frameworks to define a ground truth for a more realistic evaluation.

In conclusion, we recommend using rox for any dataset where an LOD effect can be suspected, even if the effect is not strict. The LOD assumption commonly applies to metabolomics data, as shown in this paper, but it has also been described in data with similar dropout mechanisms, such as proteomics data [32], glycomics data [33], and microbiomics data [34].

## Figures and Tables

**Table 1:** Overview of the metabolomics datasets.

Cohort	Number of Samples (Controls/Cases)	Number of Metabolites	Phenotype	Specimen	Reference
QMDiab-Plasma (HD2)	358 (177/181)	758	Type 2 Diabetes	Blood	[23]
QMDiab-Urine	360 (174/186)	891	Type 2 Diabetes	Urine	[23]
QMDiab-Saliva	330 (171/159)	602	Type 2 Diabetes	Saliva	[23]
BRCA	132 (65/67)	536	Breast Cancer	Breast Tissue	[24]
RCC	276 (138/138)	877	Kidney Cancer	Kidney Tissue	[25]
HAPO	115 (67/48)	49	Hyperglycemia	Plasma	[26]
QMDiab-Plasma Validation (HD4)	292 (137/155)	359	Type 2 Diabetes	Plasma	[27,28]

## Data Availability

All data used in this study is freely available. The git repository contains the corresponding links https://github.com/krumsieklab/rox.

## Supplementary Materials

The following supporting information can be downloaded at: https://www.mdpi.com/article/10.3390/metabo13010127/s1, Text S1: Derivation of weighted formulation; Text S2: Proof—LOD leads to $d_{b}>d>d_{1}$; Text S3: Choice of weight parameter *p* for debiasing; Figure S1: Simulation framework and small sample size; Figure S2: Simulation in multivariable setting; Figure S3: Estimating degree of LOD effect in the QMDiab data; Figure S4: Multi-Platform Validation - detailed results; Figure S5: Multi-Platform Validation-multivariable model; Figure S6: Comparable pairs with left-censoring
